# Deep Layer Aggregation Architectures for Photorealistic Universal Style Transfer

**DOI:** 10.3390/s23094528

**Published:** 2023-05-06

**Authors:** Marius Dediu, Costin-Emanuel Vasile, Călin Bîră

**Affiliations:** Faculty of Electronics, Telecommunications and Information Technology, Politehnica University of Bucharest, 060042 Bucharest, Romania; marius.dediu@stud.etti.upb.ro (M.D.); costin.vasile1003@upb.ro (C.-E.V.)

**Keywords:** deep learning, photorealistic, style transfer, deep layer aggregation

## Abstract

This paper introduces a deep learning approach to photorealistic universal style transfer that extends the PhotoNet network architecture by adding extra feature-aggregation modules. Given a pair of images representing the content and the reference of style, we augment the state-of-the-art solution mentioned above with deeper aggregation, to better fuse content and style information across the decoding layers. As opposed to the more flexible implementation of PhotoNet (i.e., PhotoNAS), which targets the minimization of inference time, our method aims to achieve better image reconstruction and a more pleasant stylization. We propose several deep layer aggregation architectures to be used as wrappers over PhotoNet, to enhance the stylization and quality of the output image.

## 1. Introduction

### 1.1. Style Transfer—An Overview

Among a wide range of rendering techniques, style transfer occupies its place through artistic and photorealistic stylization. Style transfer refers to a computer vision technique that generates an output image based on two input images (reference and style images). In this process, the content of the former is transferred to the output image using the style of the latter.

**Figure 1:**
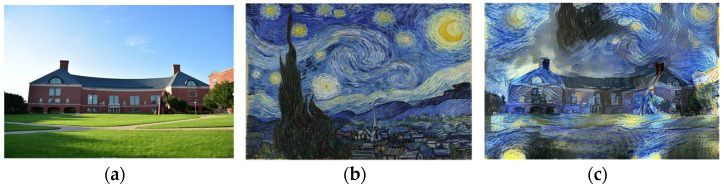
Example of artistic style transfer [3]. (**a**) content; (**b**) style; (**c**) result.

**Figure 2:**
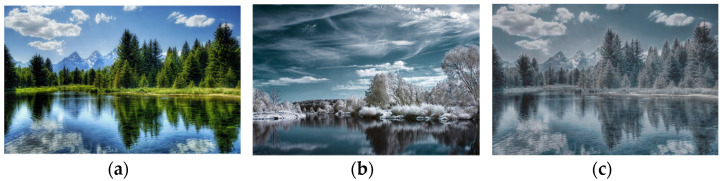
Example of photorealistic style transfer. (**a**) Content; (**b**) style; (**c**) result.

Two of the most common subfields of style transfer are artistic style transfer and photorealistic stylization [1]. Artistic style transfer offers a limited range of transfer results. It produces painterly images by being able to transfer color and patterns, introducing visible distortions when applying it to real-world images containing complex scenes [1]. On the other hand, photorealistic stylization can be seen as an extension of artistic style transfer, being able to transfer the style on a much finer level. Ideally, a photorealistic algorithm is meant to output the content image as it was captured in the same scene as the style image. Examples of both artistic and photorealistic transfer techniques are presented in Figure 1 and Figure 2. As the title suggests, this paper focuses on photorealistic style transfer techniques.

Style transfer has many applications in the arts and entertainment industries, being able to produce good aesthetic and artistic results [2]. We find it worth mentioning two of the applications that have a lot of potential:

- Photo editing and art: neural style transfer is already used in photo editing software for generating license-free artwork, art designing, fashion designing, etc. [2].
- Virtual reality: neural style transfer can generate virtual reality scenes and environments of great visual impact, much faster than conventional methods.

### 1.2. Neural Style Transfer (NST)

Given two images—content, and reference of style—neural style transfer (NST) aims to translate the former into the texture domain of the latter. To accomplish this, two main rendering steps must be performed:

- Texture synthesis: extracts the main characteristics from the input images.
- Image reconstruction: expands the synthesized content in the desired style, preserving high-level features.

From a network architecture point of view, this is accomplished using hybrids of autoencoder networks. An autoencoder is a neural network that is trained to attempt to copy its input to its output [4]: the encoder compresses the image into a “code”, a short representation of relevant features, whereas the decoder expands the “code” back into an image with similar size as the input image.

**Figure 3:**
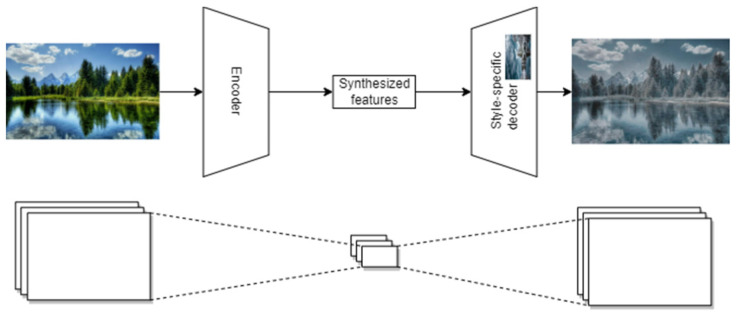
Neural style transfer architecture overview.

The neural style transfer is achieved by combining the encoder trained on the input image and the decoder trained on the style, so that the compressed input image is expanded in the style learned by the decoder (Figure 3).

#### 1.2.1. Encoder

An encoder is a feed-forward neural network used to transform its input into a compressed, latent representation, using convolutional and MaxPool layers. The output of an encoder, typically called code or bottleneck, is passed as input to the decoder in an autoencoder architecture.

J. An et al. use in [5] the pre-trained VGG-19 (Visual Geometry Group 19 Layer CNN) network as an encoder, which is a 19 layer-deep convolutional network defined by K. Simonyan and A. Zisserman in [6].

#### 1.2.2. Decoder

The decoder part plays the role of generating the final stylized image. The decoder is responsible for reconstructing the image from the encoded representation generated by the encoder, while also incorporating the style information from the style image.

It typically consists of a series of up-sampling layers that increase the spatial resolution of the encoded feature maps, followed by a series of convolutional layers that learn to generate the final image. These convolutional layers are usually similar to those used in the encoder, but with the filter sizes and number of channels reversed.

To incorporate the style information, the decoder is often augmented with additional layers that are designed to match the statistics of the style image.

Our work explores different types of deep layer aggregation techniques presented by F. Yu et al. in [7] to augment the decoder to achieve visually pleasing and semantically meaningful images.

### 1.3. Skip Connections and Deep Layer Aggregation

Skip connections are aggregation techniques used in deep neural network algorithms to improve model convergence. As the name suggests, skipping a layer in the neural network means feeding the output of one layer as the input to the next layers (instead of only to the next one) [8]. From a complexity perspective, we categorize these aggregation techniques as shallow skip connections (addition, concatenation) and deep layer aggregation (DLA).

F. Yu et al. investigate in [7] diverse ways to aggregate layers and extend shallow skip connections of previous approaches. Their experiments have shown that using deep layer aggregation achieves better performance with fewer parameters on special-purpose networks.

Our work aims to use these techniques presented by F. Yu et al. in [7], expanding the solution proposed by An et al. in [5], for better image reconstruction and more pleasant stylization in neural style transfer.

### 1.4. Motivation for Using Deep Layer Aggregation Architectures

Current style transfer methods are prone to overfitting since the decoder is trained to reconstruct the style image, which can lead to extracting spatial correlations in the style image instead of only colors and color patterns. The spatial correlations should only come from the input image. Deep layer aggregation architectures are known to reduce overfitting and might obtain better results in photorealistic style-transfer; therefore, we tested multiple aggregation techniques.

## 2. Materials and Methods

### 2.1. Deep Layer Aggregation Decoders

#### 2.1.1. Architectural Design

**Figure 4:**
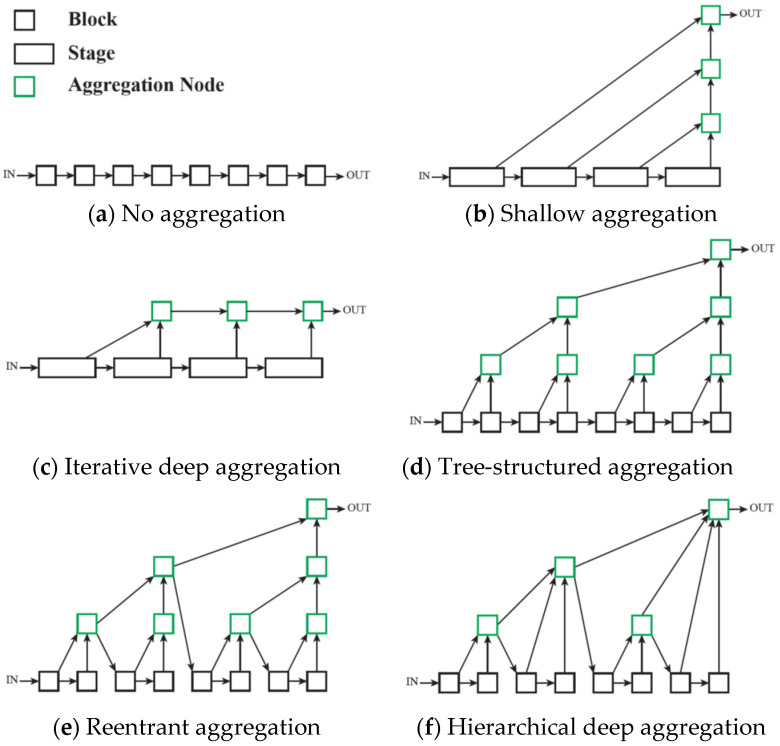
Generic photorealistic aggregation architectures, as depicted in [7]. Reprinted with permission 5541570466612 from [7], 2018, IEEE.

Each proposed aggregation strategy uses the pre-trained VGG-19 for the image classification network as the encoder and features a structurally asymmetric decoder, unlike PhotoNet, which maintains the symmetry between these two main components. Depending on the architecture’s type, deep feature aggregation is applied along the decoder to merge (concatenate) and fuse (reduce by convolutional pyramids) multi-level features in a different manner. As in [1], we used normalized skip connections at each level to directly pass extracted feature characteristics from the encoder to the corresponding decoder layer, improving the stylized image quality. Transfer modules were placed at every instance normalized skip link (INSL) stage and fuse node to enhance stylization. Each new strategy represents an end-to-end photorealistic universal style transfer solution for the given styles; hence, as in the case of [1], there was no need for pre- or post-processing operations. Furthermore, our implementation offers support for training decoders associated with new styles. Paper [7] depicts the generic photorealistic aggregation architectures shown below, in Figure 4. 

For each architecture, the fuse pyramid modules reduce the number of feature maps to the lowest value of the concatenation. Two exceptions to this rule are the bottleneck and output pyramid modules, which reduce the number of feature maps to 3 and 512, respectively. We performed up-sampling on smaller-scale feature maps before concatenation. Depending on the model used to perform style transfer, the number of pyramid modules and the values for the up-sampling factor vary accordingly.

**Figure 5:**
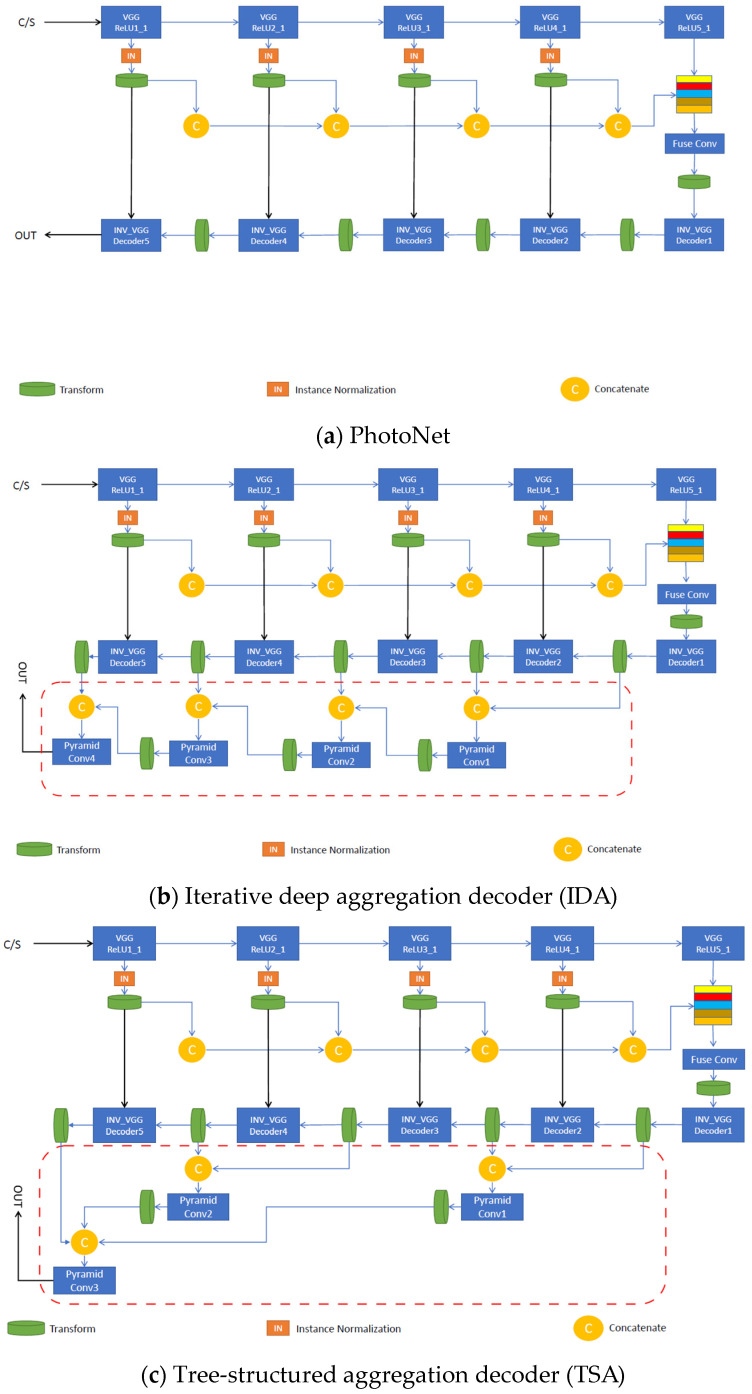

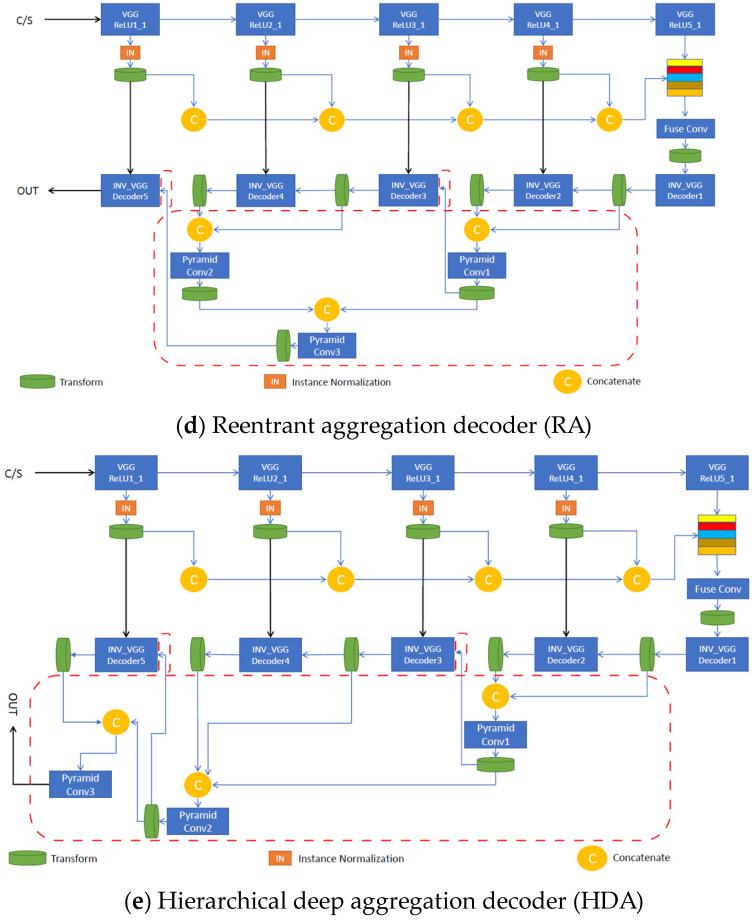
Specific implementations of photorealistic aggregation architectures, based on the generic ones: (**a**) uses deep feature aggregation and multi-stage stylization on the decoder and adds skip links to increase the image quality. (**b**) progressively merges larger features on the decoder to increase the semantic richness and resolution refinement. (**c**) better spans the features across different depths of the network using a tree-structured block. (**d**,**e**) enhance (**c**) by merging intermediate aggregations back into the network chain to improve image reconstruction and texture synthesis.

The iterative deep aggregation (IDA) decoder progressively aggregates and deepens the representation in the image reconstruction stage of the network. Aggregation begins at the smallest scale (right after the bottleneck feature aggregation module) and then iteratively merges larger scales throughout the decoder. The semantic refinement of the shallow features increases with the number of aggregation nodes. Figure 5b shows the structure of IDA.

The tree-structured aggregation (TSA) decoder aggregates hierarchically through a tree structure of blocks to better span the feature hierarchy of the network across various depths.

The reentrant aggregation (RA) decoder is a refinement of TSA that deepens aggregation by routing intermediate aggregations back into the network and improves efficiency by merging successive aggregations at the same depth.

The hierarchical deep aggregation (HDA) decoder is a much deeper, generalized feature aggregation architecture that also includes IDA, which focuses on fusing resolution and scales, whereas HDA focuses on preserving and merging features from all modules and channels [9]. We can say that HDA combines shallower and deeper layers to better learn the spatial characteristics of the features.

To keep the inference time of our network close to PhotoNet [1], we adapted these architectural designs described in [7] to fit a light universal style transfer architecture. Figure 5c–e shows the structure of the previously mentioned HDA strategies.

#### 2.1.2. Training

All aggregation decoders were trained in the same manner as PhotoNet, using the MS COCO 2014 dataset. The objective was to invert deep features received from the extraction encoder back to high-quality images. The reconstruction loss is defined as the Frobenius norm between the input and inverted output images:

$$L_{reconstruction}{=||I_{orig}-I_{recon}||}_{F}$$

where *I_orig_* represents the original image and *I_recon_* represents the image reconstructed by the decoder. In addition, a perceptual loss term is introduced to enhance the reconstruction stage of the decoder,

$$L_{perceptual}={\sum_{i=1}^{5}||\phi_{i}(I_{orig})-\phi_{i}(I_{recon})||}_{F}$$

where *Φ_i_* represents the output of the *i*^th^ stage of the pre-trained VGG-19 (*ReLU_1_1_*, *ReLU_2_1_*, *ReLU_3_1_*, *ReLU_4_1_*, *ReLU_5_1_*). This function measures high-level semantic differences between features across the network. Thus, the overall loss function,

$$L_{total}=\alpha L_{reconstruction}+(1-\alpha )L_{perceptual}$$

balances reconstruction and semantic richness through α. Training was performed removing all the transform modules from the architectural designs. During training, we set α = 0.5 to equally consider per-pixel loss and perceptual differences between input and output images.

#### 2.1.3. Expectations

By using different aggregations, it might be possible to achieve a better aesthetic effect. A better result would be comprised of improvements in one or several aspects (as detailed in Table 1):

- Improved generalization: some aggregations might be better adapted to some styles, and this would be best captured by the metric of FID score.
- Better output quality: some aggregations might be better suited to capture different aspects of the style, and this would be best captured by the metric of reconstruction error (lower value means that output image is closer to input image)
- More flexibility: some aggregations might be better suited to capturing color and spatial patterns, and this would be best captured by the metric of total variation; since the spatial coherence should come only from the input image, and the color from the style image, a very low value of total variation is a sign of content loss when doing transfer on complex images such as the ones in our dataset.

## 3. Results

### 3.1. Objective (Quantitative) Comparison

To demonstrate the image quality and pleasant photorealistic stylization of our proposed methods, we conducted a similar empirical study as in [1]. We used 40 content images (of which 38 are different), each associated with a particular style image. We used WCT (whitening & coloring transform) [10] as a transform operation across decoders as shown in Figure 5.

A Fréchet inception distance (FID) [11] was used to outline the similarity in style between the content dataset and the resulting stylized dataset, respectively. A lower FID score denotes higher similarity and therefore better stylization. The FID is experimentally proven in [10] to assure a consistency between disturbance level and human judgment in various situations (gaussian noise, gaussian blur, black rectangles, swirl, salt and pepper noise, ImageNet contamination).

The discrete total variation (TV) [12] was computed to show the level of detail from the transferred image. A very small value of TV represents a serious drawback: distortion and noise are not penalized as intuitively as they should be.

We also measured the feature inversion capabilities of each decoder by calculating the reconstruction error between the reconstructed image and the corresponding original image as follows,

$$\varepsilon_{reconstruction}=\sum_{i=1}^{N}{\left|\left|I_{orig}-I_{recon}\right|\right|}_{F}/N$$

where *N* represents the number of images from our content dataset.

The FID and TV were computed on 768 × 512 transferred images, while the reconstruction error was calculated using pairs of 512 × 512 images.

**Figure 6:**
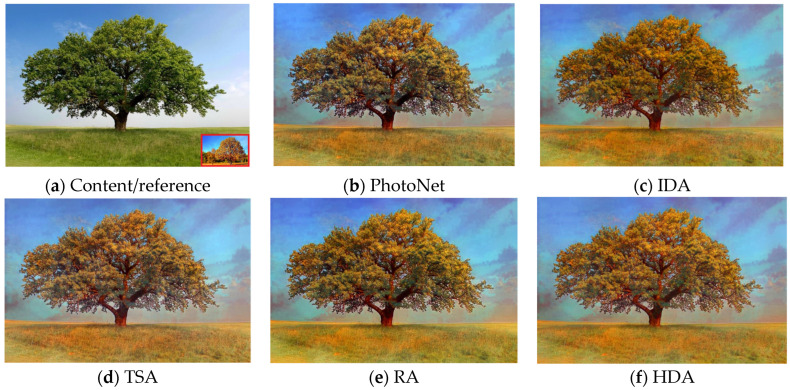
Aesthetic comparison of photorealistic style transfer. (**a**) is the content image; the reference of style is shown in the bottom-right corner of (**a**). (**c**) removes stylization artifacts from (**b**) and better preserves the image semantics. (**d**) combines texture from different layers and focuses mainly on style synthesis. (**e**,**f**) use (**d**) as a backbone and provide more localized stylization by feeding fuse pyramid modules back into the main decoder.

However, by analyzing the way in which our strategies transfer texture from the style reference to the content input using Figure 6 in correlation with Table 2, we can state that a higher TV score does not necessarily suggest a better quality or detail richness, but can denote poor semantic consistency in the output image by means of scattered stylization. In this respect, if we visually compare Figure 6b with Figure 6f, which have a TV score of 6957.632 and 4891.987, respectively, it is quite clear that the latter exhibits a more compact and semantically accurate stylization, even though its TV score is lower. In addition, Table 2 shows that RA achieves the lowest reconstruction error, while IA thrives in terms of stylization by obtaining the lowest FID score. We trained the decoders for 5 epochs and discovered that depending on the aggregation strategy, the best results for each were obtained earlier or later in the training process. For example, IA, TSA, and HDA required more epochs to achieve better image reconstruction and lower FID, while PhotoNet and RA minimized the reconstruction error and FID score earlier.

### 3.2. Subjective Comparison

**Figure 7:**
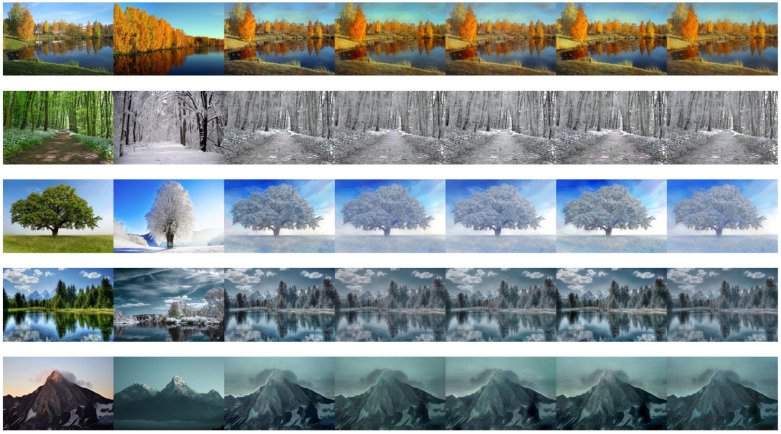
Subjective comparison samples (downsized to fit the page width of this paper). The 34 human subjects had to choose the best style transfer for each of the five different experiments. From left to right for each experiment, we have: the content image, the style image, and then, the style transfer results: PhotoNet, IDA, TSA, RA, HDA.

Out of a total of 170 votes performed on five different samples, RA was the most preferred photorealistic style transfer approach according to Table 3. Figure 7 below, shows the five different samples (vertically stacked).

### 3.3. Comparison between Expected Results and Measured Results

We compared the expected results with the measured results (see Table 4), and we found that the aggregation technique of IDA was the most unpleasing version for the subjective results, while simultaneously having a low score for the TV metric, which hinted at a loss of detail.

Regarding the TSA and HDA, the results were similar to PhotoNet (marginal improvements or setbacks).

The RA aggregation technique showed improved (low) reconstruction error and was the most pleasing version in the subjective evaluation, when compared to PhotoNet or all other aggregation techniques.

### 3.4. Computational Time Comparison

We computed both the inference and training time for each architecture and show the results in Table 5. The evaluation was executed on the same platform in all cases, which has as the main training hardware device an NVIDIA Titan RTX TU102 GPU with 24 GB of RAM. Training was performed using resized 512 × 512 images from the MS COCO 2014 dataset, while inference processes resized 768 × 512 images from our content and style dataset, respectively. The differences in both time metrics being in favor of PhotoNet are acceptable, given the extra aggregation layers used in our proposed decoders.

## 4. Conclusions

Aggregation is an important aspect, not only for classification or high-resolution network architectures, but also for photorealistic style transfer purposes. By addressing deep aggregation in the context of photorealistic style transfer, we demonstrated that different aggregation approaches led to different stylization capabilities.

Compared to plain PhotoNet, our experiments with extra aggregation techniques led to longer training and inference times (as expected, because we added extra layers, which have more weights to train and infer) and for RA (reentrant aggregation decoder), it led to better subjective results, proving, thus, to be a more pleasing stylization result for humans. This result was also indicated by the reconstruction error, which was lower than when using the PhotoNet alone. We checked results of total variation (TV) and the Fréchet inception (FI) scores and they were similar to PhotoNet scores. 

As future work, we would like to repeat the experiment on grayscale/infrared-spectrum images, and on higher-definition images when an NVIDIA GPU such as the RTX H100 with 80 GB of RAM memory becomes available.

Other areas where these NST decoders might be tested are data augmentation of medical data (see [13,14]) and material translation [15]; basically, any domain where we can objectively measure the impact of various decoder architectures, is a candidate for using the work and results of this paper.

## Figures and Tables

**Table 1:** Expected results when using IDA/TSA/RA/HDA aggregation techniques along with the metric that should measure properly the expected results.

Architecture	Short Description of Architecture	Expected Improved Aspect	Metric for Improved Aspect
IDA	Iteratively refines the output image by adding details at multiple scales. Should gradually improve image quality by adding fine details and texture. Decreased generalization might happen due to overfitting.	Improved quality Increased flexibility Decreased generalization	Reconstruction Error Total Variation FID score
TSA	Organizing aggregations in a tree structure, each node capturing a different aspect of the style. Decreased generalization might happen due to overfitting.	(Potentially) improved quality Decreased generalization	Reconstruction Error FID score
RA	Reentrant aggregation involves a recursive process of combining multiple layers of feature maps from different layers of the convolutional neural network, allowing for a more detailed and fine-grained control over the style transfer process (by having a spatial memory).	(Potentially) improved quality Increased flexibility	Reconstruction Error Total Variation
HDA	Multiple layers are combined hierarchically to produce output image: should be able to capture both local and global features of the style and content.	Improved quality Improved generalization	Reconstruction error FID score

**Table 2:** Objective (quantitative) results. RA achieved the lowest image reconstruction error, while PhotoNet and IDA obtained the highest TV and lowest FID, respectively. We trained our decoders for multiple epochs to show that no exact correlation can be established between these three metrics of interest. Each architecture can be considered a specialized style transfer method, depending on its main capability (e.g., better semantic richness, higher image quality, etc.).

Architecture	Epoch	Reconstruction Error (Lower Is Better)	Total Variation Score (TV) (Very Low Is Bad)	Fréchet Inception Distance Score (FID) (Lower Is Better)
PhotoNet	1 2 3 4 5	102,211.791 98,190.635 **94,087.596** 108,593.92 102,166.251	8189.258 **8325.533** 7271.771 7393.16 6957.632	163.79 **160.23** 160.28 161.92 163.71
IDA	1 2 3 4 5	115,927.932 93,623.708 105,055.555 **92,252.028** 89,256.107	4205.513 4375.632 **4618.789** 4340.921 4291.648	161.45 159.38 160.53 **158.28** 160.04
TSA	1 2 3 4 5	101,494.835 112,259.242 107,383.347 **91,525.225** 117,641.887	5037.395 4896.852 5072.647 **5243.896** 5089.479	162.96 160.98 161.65 **160.3** 160.32
RA	1 2 3 4 5	**73,907.531** 131,479.38 113,357.705 106,816.984 90,576.84	7244.274 **8140.81** 7048.334 7184.867 7026.428	164.87 163.04 **160.78** 160.99 160.85
HDA	1 2 3 4 5	124,712.988 108,710.381 113,971.76 **97,956.429** 99,175.09	4810.022 4815.858 4791.003 **5077.516** 4891.987	162.31 161.48 **160.49** 163.18 161.03

**Table 3:** Subjective results. A total of 34 people were asked to choose their preferred photorealistic version resulting from the combination of several content and style images. Out of a total of 170 votes performed on 5 different samples, RA is the most preferred photorealistic style transfer approach, adopting an equilibrium between image reconstruction and stylization.

Architecture	PhotoNet	IDA	TSA	RA	HDA
Preference	22.35%	7%	12.95%	41.8%	15.9%

**Table 4:** Comparison between expected result and measured result of IDA/TSA/RA/HDA aggregation techniques.

Architecture	Expected Impact	Metric for Improved Aspect	Result Matches the Expected Result?
IDA	Improved quality Increased flexibility Decreased generalization	Reconstruction Error Total Variation FID score	Yes, but marginally better than PhotoNet Low value, could be bad: check subjective results No, but marginally better than PhotoNet
TSA	(Potential) improved quality Decreased generalization	Reconstruction Error FID score	Yes, but marginally better than PhotoNet No, but similar to PhotoNet
RA	(Potential) improved quality Increased flexibility	Reconstruction Error Total Variation	Yes, improved Similar to PhotoNet
HDA	(Potential) improved quality Improved generalization	Reconstruction error FID score	No, but marginally worse than PhotoNet No, but similar to PhotoNet

**Table 5:** Computational evaluation. Both inference and training time are higher than PhotoNet’s for our strategies on behalf of the extra layers added. The time is calculated as the average time to transfer a style from the style dataset to the content dataset.

Architecture	PhotoNet	IDA	TSA	RA	HDA
Inference (s)	1.02	1.34	1.65	1.45	1.45
Training (min)	128	135	142	135	136

## Data Availability

Data available in a publicly accessible repository that does not issue DOIs. This data can be found here: https://gitlab.upb.ro/research/styletransfer/ustusingdla.
